# A machine learning tool for interpretation of Mass Transport Deposits from seismic data

**DOI:** 10.1038/s41598-020-71088-6

**Published:** 2020-08-24

**Authors:** Priyadarshi Chinmoy Kumar, Kalachand Sain

**Affiliations:** Wadia Institute of Himalayan Geology, 33 GMS Road, Dehradun, 248001 India

**Keywords:** Geophysics, Solid Earth sciences

## Abstract

Machine learning is a tool that allows machines or intelligent systems to learn and get equipped to solve complex problems in predicting reliable outcome. The learning process consists of a set of computer algorithms that are employed to a small segment of data with a view to speed up realistic interpretation from entire data without extensive human intervention. Here we present an approach of supervised learning based on artificial neural network to automate the process of delineating structural distribution of Mass Transport Deposit (MTD) from 3D reflection seismic data. The responses, defined by a set of individual attributes, corresponding to the MTD, are computed from seismic volume and amalgamated them into a hybrid attribute. This generated new attribute, called as MTD Cube meta-attribute, does not only define the subsurface architecture of MTD distinctly but also reduces the human involvement thereby accelerating the process of interpretation. The system, after being fully trained, quality checked and validated, automatically delimits the structural geometry of MTDs within the Karewa prospect in northern Taranaki Basin off New Zealand, where MTDs are evidenced.

## Introduction

Mass Transport Deposits (MTDs), occurring in different tectonic and depositional settings, are defined as gravity induced slope failure deposits that include creeps, slides, slumps and debris flows^1−6^. These deposits are internally deformed and associated with variable shape and size. During slope failure, masses tend to flow downslope over a shearing surface, called the basal shear surface (BSS) that forms the base of the MTDs. BSS preserves the record of all erosional and deformational activities experienced by these deposits or masses during their translation. Their interpretation is crucial, as such deposits during translation over the instable slope may lead to several catastrophic submarine events e.g., landslides, tsunamis, avalanches and thus possess precursory threats for subsea installations^7−15^.

Several authors^16−25^ attempted to study the MTDs in order to understand their evolution, geomorphic character and possible trigger mechanisms responsible for slope failure. The use of modern techniques e.g., reflection seismic (2D/3D), side scan sonar, bathymetry etc. added value for their detailed investigation. In reflection seismic, the MTDs are first identified by mapping their top and BSS, and then interpreted from cross-sections and attribute maps^23−27^. For this, the seismic attributes such as the root-mean square (RMS) amplitude, dip magnitude and coherency have been used for the interpretation of this geologic feature^23−26^. Though the single attribute technology has been successful in interpreting MTDs from seismic data, several authors^28−29^ demonstrated the downside of such approach, where a single attribute hardly ever responds to a particular geological target *(see sections “Initial Interpretation” and “From Seismic Attributes to Meta-attributes” in the Supplementary Note for detailed explanation).*

**Figure 1:**
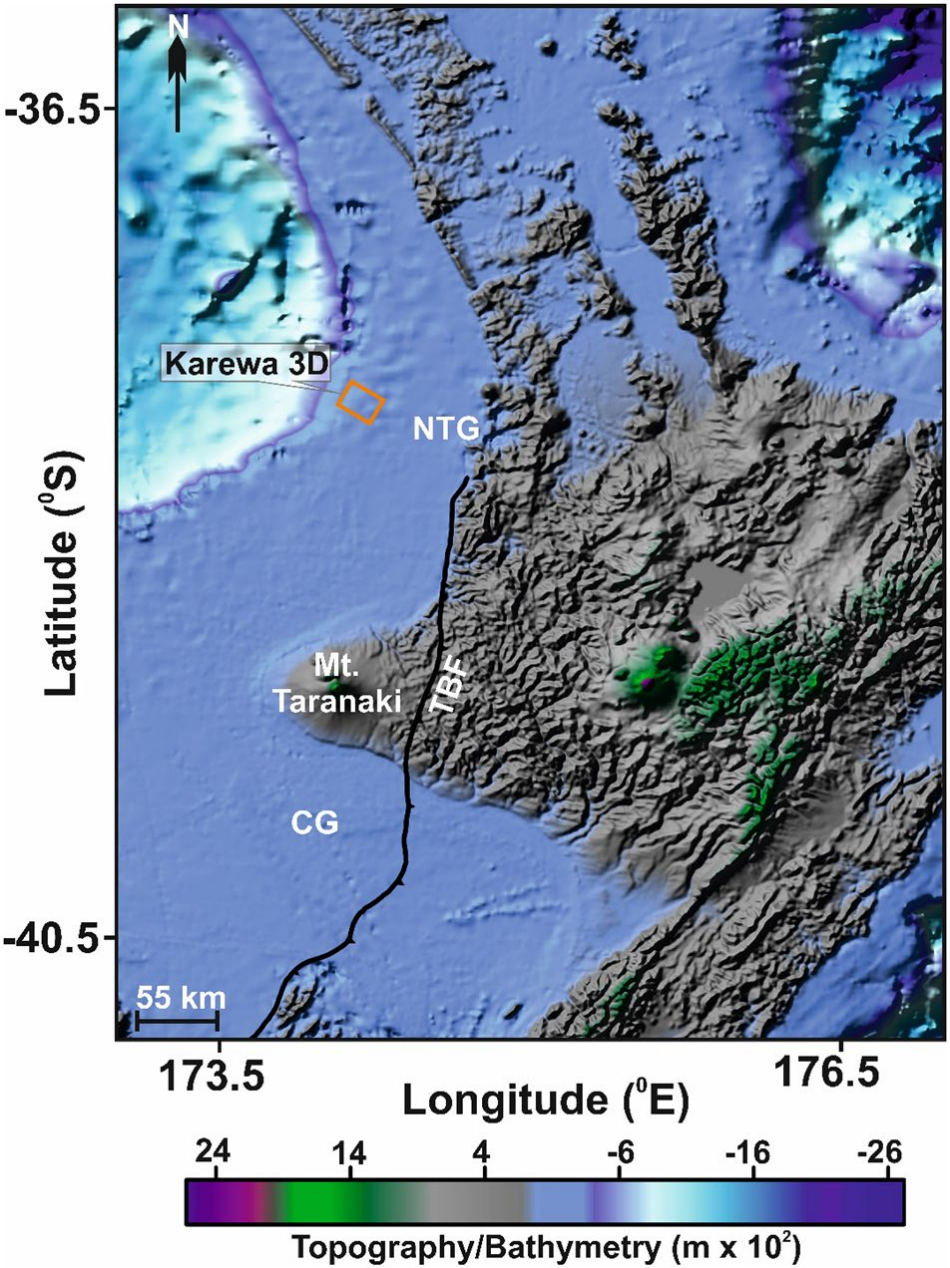
Location of the study area in the northern Taranaki Basin, offshore New Zealand. The Karewa 3D prospect, shown by orange rectangular box, lies close to the Northern Taranaki Graben (NTG). (Adopted from the Gridded Bathymetry Chart of the Oceans (GEBCO) compilation group, 2019). CG—Central Graben; TBF-Taranaki Boundary Fault.

The Taranaki Basin (TB) is a well-known hydrocarbon producing region off New Zealand^30^ and mainly lies to the west of the North Island (Fig. 1). The basin is ~ 60 km wide and extends ~ 350 km in the NNE direction from south of the Taranaki peninsula to the offshore west of Auckland^31^. The basin forms part of the overriding Australian plate and lies about ~ 400 km west of the Hikurangi Trough, where the Pacific plate is subducted^32−33^. Tectonic evolution of the basin includes extension during the Late Cretaceous to Early Eocene, followed by compression during the Late Eocene and back-arc extension from the Late Miocene to Recent^32^. Towards the end of Miocene, the basin accumulated large sediment influx, known as the Giant Foresets, deposited in shelf to basin succession^27,33−35^. MTDs are widespread within this basin and have been recognised throughout the offshore in TB. Among these deposits, a submarine MTD, called the Karewa MTD (~ 25 km long and 4 km wide) lies within the northern TB offshore. Panpichityota et al.^36^ used manual method coupled with attribute analysis for mapping the Karewa MTD with a view to understand their geometric relation with the Karewa fault within the study area.

The present study attempts to demonstrate a semi-automatic approach for the interpretation of Karewa MTD within the Karewa 3D prospect (Fig. 1) from reflection seismic data based on artificial neural networks. In this process, a human analyst is tasked with analysing a small part of the data, which the algorithm uses as input in order to complete the rest of the analysis automatically—therefore accelerating the process. The research aims to design a workflow for the computation of a meta-attribute by amalgamating a number of seismic attributes sensitive to MTD. To achieve this, the time-migrated seismic data is structurally conditioned *(see the section “structural enhancement” in the Supplementary Note for detailed explanation)* to make it free from noises. Seismic attributes at randomly selected few example locations labelled by an interpreter are used to train the system (*see the section “the MTD cube meta-attribute*” *in the Supplementary Note for detailed explanation*). Such neural training outputs a hybrid attribute, called the MTD cube or MTDC meta-attribute (defined for the first time) that conspicuously delimits the geometry and distribution of Karewa MTD and augments interpretation of entire reflection seismic data with a much reduced human intervention.

### Results

**Figure 2:**
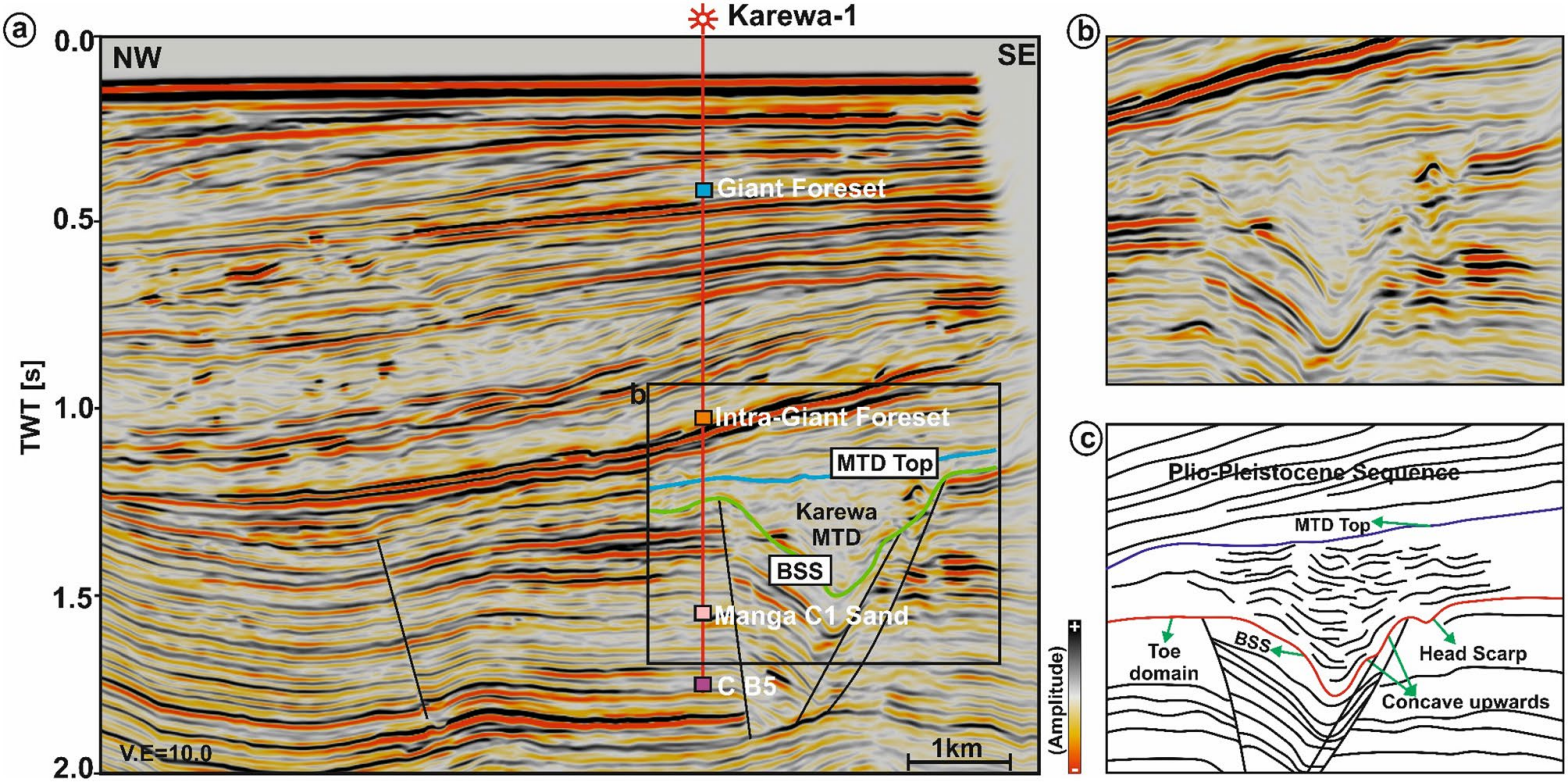
(**a**) Conditioned seismic section for line IL 1,278 demonstrating the subsurface architecture of the Karewa prospect, drilled by well Karewa-1 which is terminated at C B5 strata. The eastern part of the prospect is dominated by the Karewa MTD; (**b**) Zoomed view of the MTD zone, as marked by black rectangle in (**a**); (**c**) Interpreted sketch of corresponding Karewa MTD, bounded by the PPS on the top, and basal shearing surface (BSS) at the bottom exhibiting concave upward geometry. The head domain lies on the eastern part and toe domain towards the western part of the MTD zone, characterized by deformed sediment units and internally chaotic reflections.

**Figure 3:**
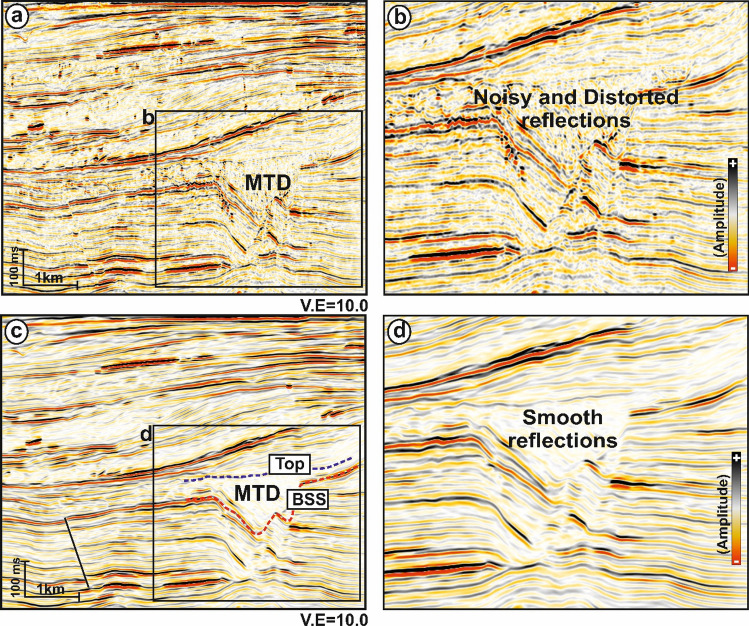
(**a**) Original time migrated seismic section for line IL 1,124 from the Karewa prospect; (**b**) zoomed view of the MTD zone, marked by black rectangle in (**a**). The MTD zone is mixed up with noises and distorted reflections. (**c**) Dip Steered Median Filtered (DSMF) conditioned seismic section for the same line (IL 1,124); (**d**) zoomed view of the MTD zone, marked by black rectangle in (**c**) showing improved and smoothed image free from noises.

**Figure 4:**
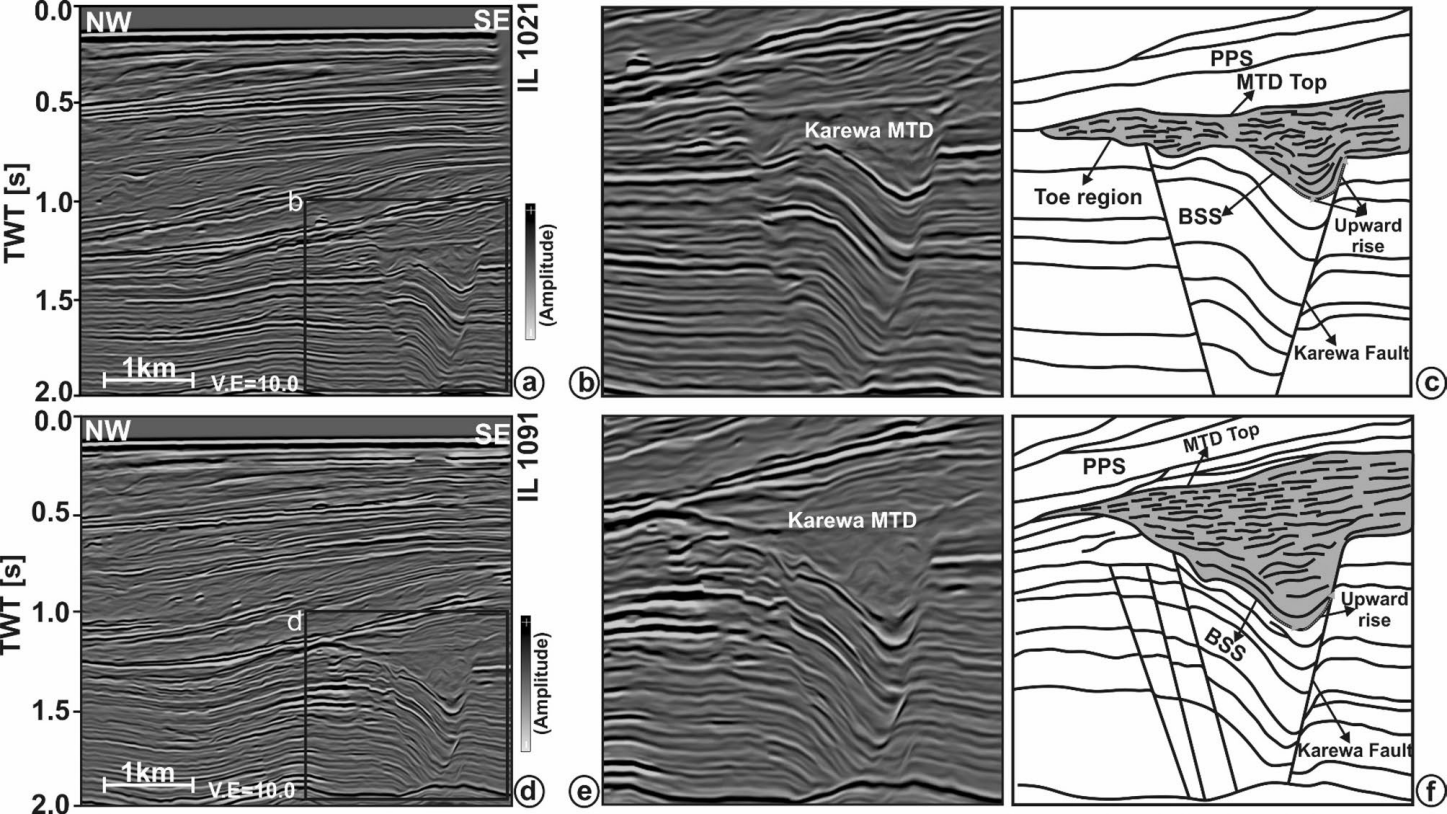
(**a**) Conditioned seismic section for line IL 1,021 demonstrating the subsurface architecture of the Karewa prospect; (**b**) Zoomed view of the MTD zone, marked by black rectangle in (**a**); (**c**) Interpreted sketch of corresponding Karewa MTD; (**d**) Conditioned seismic section for line IL 1,091 demonstrating the subsurface architecture of the Karewa prospect; (**e**) Zoomed view of the MTD zone, marked by black rectangle in (**d**); (**f**) Interpreted sketch of corresponding Karewa MTD. The sections show that the Karewa MTD is fault bounded both on the head wall and on the toe domain. The BSS of MTD rises upward along the Karewa fault towards the eastern part. The top of MTD runs almost parallel to the overlying PPS.

**Figure 5:**
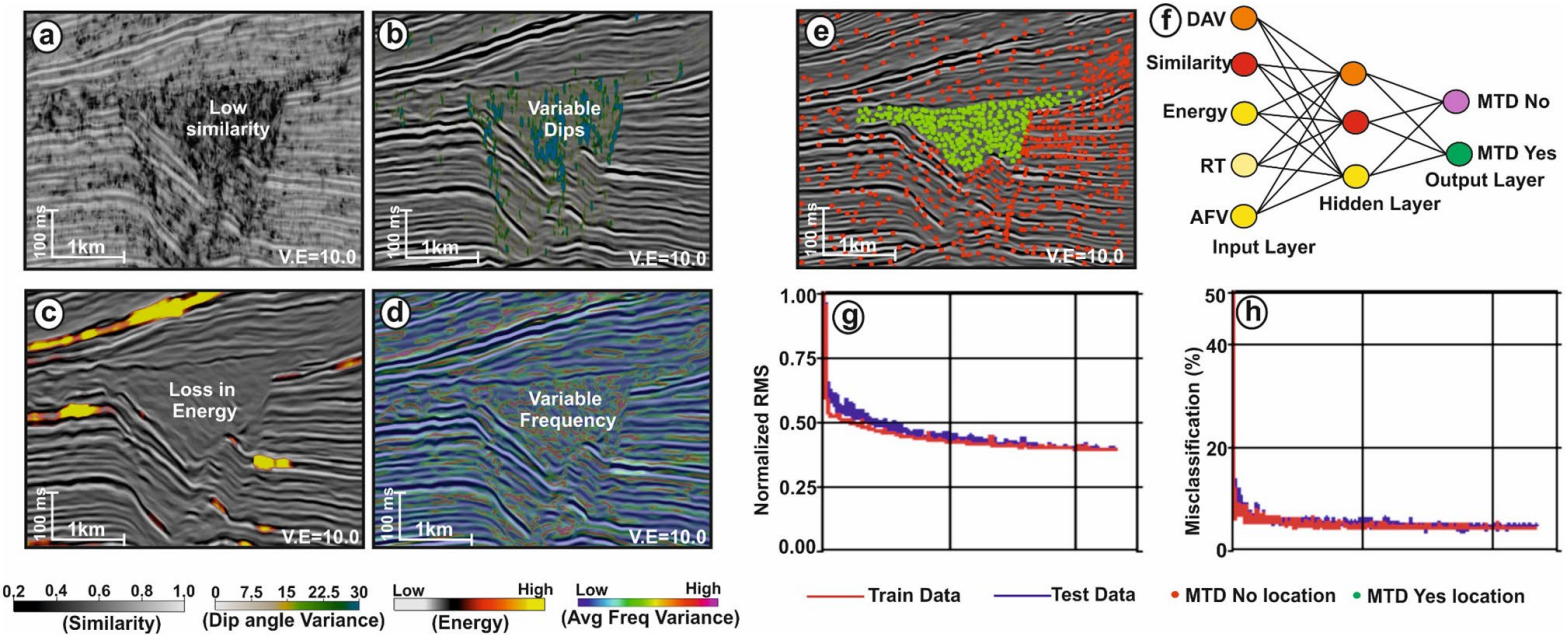
Computed seismic attributes (**a**) similarity, (**b**) dip angle variance (DAV), (**c**) energy, (**d**) average frequency variance (AFV) within the Karewa MTD, associated with low similarity, variable dips, loss in energy and variable frequency content. Attributes are displayed by co-rendering with the seismic section for line IL 1,124; (**e**) Picked locations of MTD-yes (green dots) and MTD-no (red dots) locations on a portion of seismic line (IL 1,124) as an example; (**f**) A fully connected MLP network in color code showing relative contribution of nodes (light yellow (least contribution) through orange to red (highest contribution)); (**g**) Normalized RMS error, and (**h**) Misclassification (%) for the training (red) and testing (blue) data sets respectively. RT: Reference Time.

The present work mainly focuses on the interpretation of reflection seismic data in the shallow section (Fig. 2) within which the Karewa slump zone occurs. The slump zone is overlain by the Plio-Pleistocene Sequence (PPS) to the recent sedimentary deposits. Moreover, the slump zone is bounded by the Karewa fault on the eastern part of the prospect. The Karewa prospect is drilled up to a depth of 2,215 m by the well Karewa-1^37^, which has penetrated the Pliocene Manga C1 sand-dominated geologic formation associated with elevated amplitudes on seismic data (Fig. 2). The original seismic cube is prone to have several noises that mask the Karewa MTD disturbing its visualization and hence interpretation (Fig. 3a,b). Structural conditioning of the data enhances the targeted zone by removing noises and distorted reflections (Fig. 3c,d). Internally, the Karewa MTD is observed to be structurally deformed (Figs. 2,3c,d). The top of the MTD runs more or less parallel to the upper bounding PPS. The BSS exhibits concave geometry with the limbs of shearing surface transgressing upwards on the eastern part. The headwall and toe domains of MTD lie on the eastern and western part of the study area respectively (Fig. 4). The translation domain i.e. the main body of the Karewa MTD is bounded by a set of fault systems that are antithetic to the Karewa fault lying on the eastern part (Fig. 4b,c,e,f). The shearing surface exhibits an upward rising geometry along the Karewa fault. Seismic attributes aid for interpretation by capturing structural responses of the MTD, which is associated with discontinuous reflections as seen by low similarity attribute (Fig. 5a) and variable dips as implied by dip variance attribute (Fig. 5b). The MTD is not only discontinuous in nature but also internally deformed and contains rafted sediment units, resulting into the loss of energy (Fig. 5c) and frequency (Fig. 5d).

**Figure 6:**
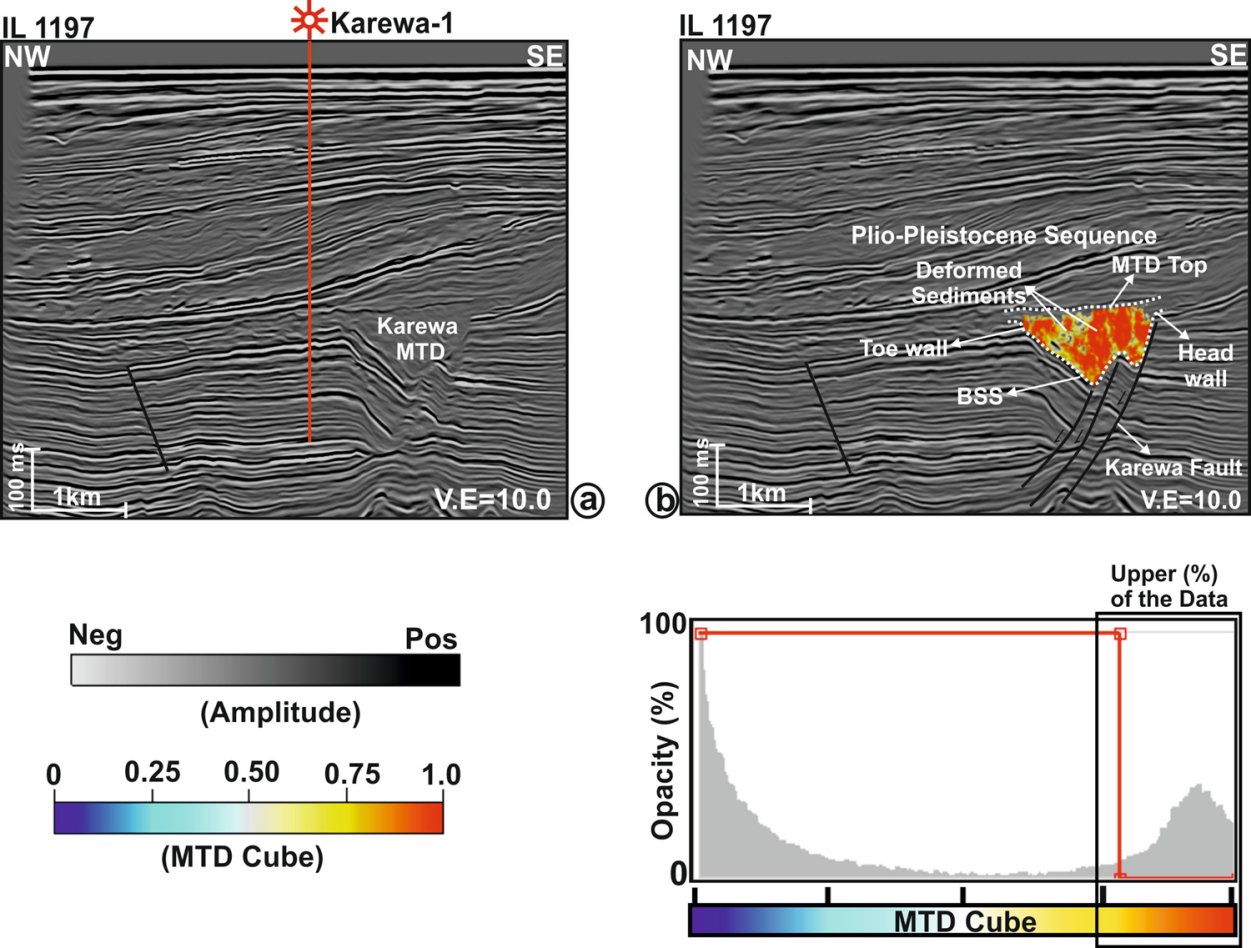
(**a**) Seismic section for line IL 1,197 demonstrating the Karewa MTD in the eastern part; (**b**) The same section, co-rendered with the machine generated MTD cube meta-attribute. A pastel color scale is used to display the meta-attribute, where red signifies the highest probability of MTD.

**Figure 7:**
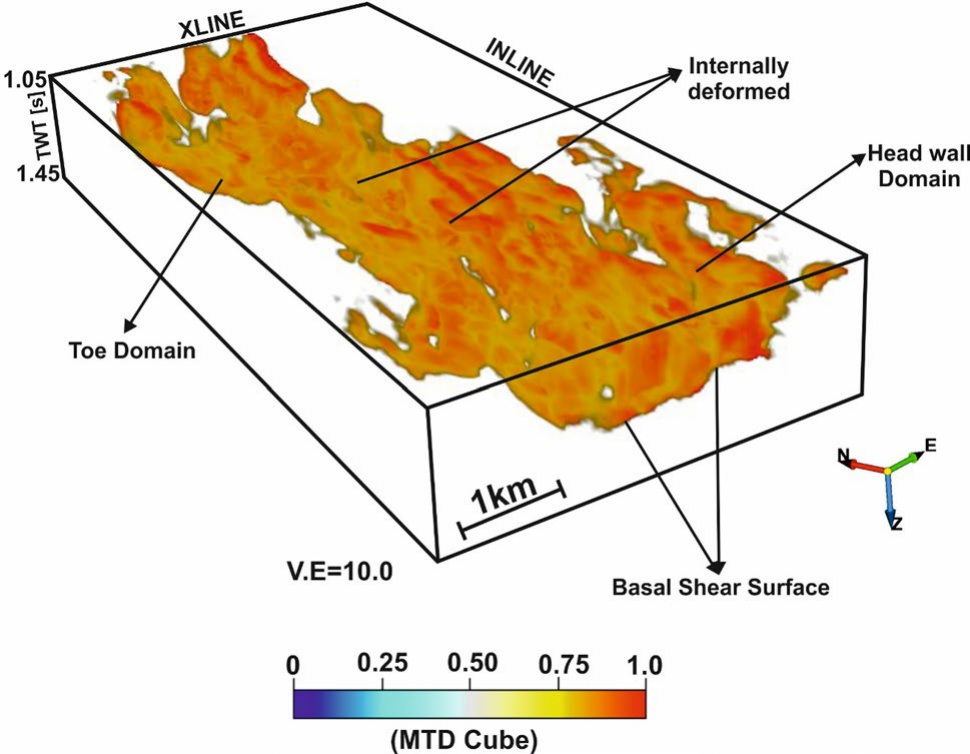
3D structural geometry and distribution of Karewa MTD, as brought out by the MTD cube meta-attribute from seismic data.

Based upon seismic characteristics and properties, a human analyst picks up by hand and labels the MTD-yes and MTD-no locations (Fig. 5e) over the randomly selected few xlines and inlines from the 3D seismic cube. The MTD-yes and MTD-no targets/objects are assigned with 1 and 0 according to the binary classification rule. The hand-labelled data is split into 70/30% chunks for training and testing respectively. Using these small segments of data, a fully connected multi-layer perceptron (MLP) network (Fig. 5f) is iteratively trained by a feed forward process using seismic attributes (similarity, dip angle variance (DAV), energy, frequency variance and reference time). This results into a minimum normalized root-mean square (nRMS) error (Fig. 5g) and low misclassification percentage (Fig. 5h) for both the train and test data sets *(see sub-sections “Attribute Selection”, “Example Locations (Train/Test Data)” and “Neural Network Analysis” in the Supplementary Note for detailed explanation).* It is observed that the nRMS of 0.3 and 0.45, and minimum misclassification of 6.05% and 8.02% are achieved after 25 iterations for the train and test data sets respectively. The relative contribution provided by the seismic attributes, while training the system, is given in Table 1. This demonstrates that the similarity attribute offers the maximum contribution to the neural training followed by the DAV, energy, reference time and frequency variance attributes. Once the system is fully trained, quality checked and validated, the network is run over the entire 3D volume. The machine automatically predicts the MTDC meta-attribute, which is a probability cube with values ranging between 0 and 1 (bottom panel of Fig. 6). The values closer to 0 show the least probability of MTD and those closer to 1 indicate the highest probability for the occurrence of MTD. An optimum color scale (i.e., pastel) is used in such a way that the maximum probability is displayed, visualized and those pertaining to the minimum probability is made transparent. For automatic delineation of MTD by machine, we have fixed the threshold value of 0.75 probability from the final outcome. To validate this outcome, we have made a comparison between the conditioned seismic section (Fig. 6a) and the same section co-rendered with machine generated MTDC meta-attribute (Fig. 6b) along another random line, which was not considered while training the system. This shows that the predicted meta-attribute has efficiently captured the Karewa MTD. Moreover, the meta-attribute has also arrested the lateral extension of MTD in the headwall and toe domains lying to the eastern and western part of the study area. It is to be noted that Figs. 2 and 6 are on different lines. The base of the MTD resembles a w-shape structure with the BSS rising upward (Fig. 6b). The final outcome or the MTDC meta-attribute (Fig. 7) has clearly brought out the structural elements and NW–SE elongated 3D geometry of MTD in the Karewa prospect. The MTD covers an area of ~ 20 km^2^ and is dominant in SE of the Karewa prospect.

**Table 1:** Relative contribution of each individual seismic attribute to the neural training.

Seismic attributes	Weights
Similarity	96.8
DAV	87.8
Energy	78
Reference time	75
Average frequency variance	62

**Table 2:** Computation time required for executing the process.

Data (individually when used for interpretation)	Computation time (s)	Neural network (Amalgamation of several inputs)	Computation time (s)
Steering seismic	59,194	NN for MTD Cube	21
DSMF seismic	66,637		
Similarity	8,516		
DAV	6,585		
Energy	3,482		
Average frequency	2024		

**Figure 8:**
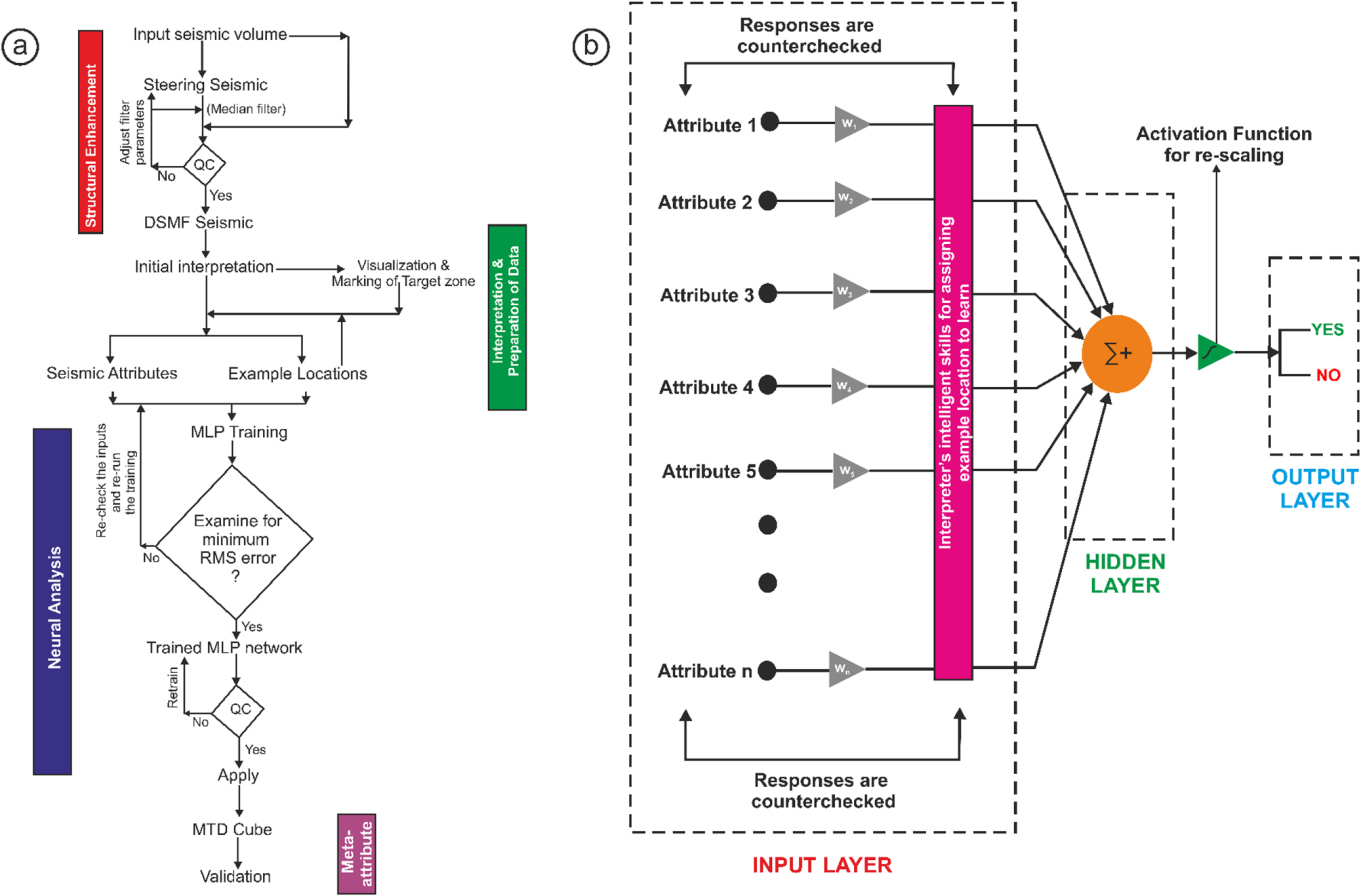
(**a**) Workflow and (**b**) the Neural Network architecture used in the present approach.

In Table 2, we present the fastness of this approach as compared to the computation of individual attributes, which is carried out on routine basis for the interpretation of MTD from seismic data. It is observed that the meta-attribute approach is much faster as compared to the conventional practices. The entire work is summarised by a flow diagram in Fig. 8.

## Discussion

Interpretation of MTD or similar geologic features from reflection seismic data becomes tedious and erroneous when they are contaminated with random noises. We have structurally enhanced the data (Fig. 3) in the first phase and then moved to the next step for automatic interpretation of data., Panpichityota et al^36^ attempted manual approach to map the MTD in understanding its relation within the bounded fault system through individual attributes. However, the characteristic of a single attribute with respect to a particular target from the surrounding may be questionable^28−29^. To circumvent such a perplexing situation, we have developed a semi-automatic approach for delineating the MTD geometry from 3D seismic data by designing a workflow and computing a new meta-attribute.

It is crucial to mention that the success of such interpretation depends on how best the system is trained by a human analyst to distinguish the targeted geologic features and their characteristic properties from the surrounding sedimentary units and other artefacts^28−29^. The MTD has been defined from seismic data by computing a hybrid- or meta-attribute, which has been generated by combining a set of other seismic attributes that are specific to MTD using a machine learning approach. Though the outcome is generally validated with the existing geology or well information or available literatures or petroleum reports^36,38^, the performance of the neural model is checked here by visual inspection. Comparison of seismic section (Fig. 6a) over a line that was not chosen during training with the same section clipped with the predicted outcome (Fig. 6b) of ANN shows that the MTDC meta-attribute has captured the MTD structure quite accurately. This shows the efficacy of automatic delineation and structural interpretation of MTD from 3D seismic data and validates the design of neural network. By scanning over the entire seismic volume, the resulting MTDC meta-attribute has prominently delimited the internal structure, extension and distribution of MTD in the Karewa prospect (Fig. 7).

Like many other techniques, this approach may also fail if the data is too noisy to remove and/or if the network is not correctly trained.

### Conclusion

The major conclusions drawn from this study are summarized below:

- The noise in original time migrated seismic data in the Karewa prospect has been removed considerably by a dip steered median filter (DSMF) and subsurface image of mass transport deposit (MTD) has been enhanced.
- This has made possible to utilise the data for automatic delimitation of 3D structural geometry and extent of MTD in the Karewa prospect.
- A new workflow has been designed by which a set of individual seismic attributes or responses of MTD has been combined into a hybrid, defined as MTDC meta-attribute based on ANN approach.
- The MTDC meta-attribute, first of its kind, has been very efficient in capturing the 3D structural elements of MTD in Karewa prospect from reflection seismic data.
- The study brought out 3D structural configuration of NW–SE elongated MTD covering an area of ~ 20 km^2^. The MTD is dominant in SE of the Karewa prospect, and internally deformed with sheared base.
- This approach is fast and semi-automatic that can be used not only for advanced interpretation of MTDs from world-wide sedimentary basins but can be suited for the interpretation of any other complex subsurface feature from 3D seismic data.

### Data and research method

The data used for this research includes a time migrated 3D seismic data that consists of 393 inlines (Line no. 1000 to 1,393) and 2000 xlines (Line no.2800 to 4,800) over the Karewa prospect in the northern offshore TB (Fig. 1). The seismic data, which was acquired by PGS M/V Orient Explorer, covers an area of ~ 122 km^2^. Additional acquisition parameters include bin spacing of 25.0 m × 12.5 m (inl/xrl), 4 ms sampling interval and 5 s record length. The primary goal of the 3D seismic survey was to accurately image the Karewa structure with a view to provide high quality reservoir property volumes^38^. The acquired data have been processed using routine work flows that includes reformatting, amplitude matching, navigation merging, spherical divergence, swell noise attenuation, ensemble balancing, tidal statics, tau-p deconvolution, radon demultiple followed by Kirchhoff time migration to obtain 3D subsurface image of the Karewa prospect. The data are displayed using SEG American polarity convention where an increase in acoustic impedance is represented by a peak (positive amplitude-black on seismic sections). For a dominant frequency of 40 Hz within the Karewa MTD and assuming the sediment velocity of 1,800 m/s, the value of $\lambda /4$ i.e., the limit of vertical resolution is 10 m.

The method adopted here is a step-by-step approach (Fig. 8a) consisting of (1) Structural enhancement of seismic data; (2) Computation of suitable seismic attributes and selection of training/testing locations; (3) Setting up a logical neural network to compute the MTDC meta-attribute; and (4) Validating the MTDC meta-attribute outcome by clipping the result over a few un-interpreted seismic sections that show capturing of Karewa MTD quite accurately.

### Structural enhancement of seismic data (see the section “Structural Enhancement” supplementary note for detailed explanation)

Structural enhancement of the Karewa seismic cube is performed using a structure oriented filter (SOF) that utilizes pre-computed dip-azimuth volumes^28−29,39^ (known as the steering cube) to steer the data in the direction of local dip of the seismic events^40^. The key objective behind this filtering is to differentiate between the dip-azimuth of the seismic reflectors and the overlying noises^28,29,41^. Such filtering does not only help in removing the random noise from the data but preserves the amplitude content and enhances the lateral continuity^39,42,43,44^. A statistical filter, called as dip steered median filter (DSMF), is applied to the seismic cube using the pre-processed steering cube, with a 3 × 3 median filtering step-out. This results into an enhanced DSMF seismic cube, which, after detailed quality check, is used for attribute extraction, selection of training/testing locations and setting up neural network (NN) for interpretation (Fig. 8).

### Seismic attributes and their selection (see the sub-section “Attribute Selection” in the supplementary note for detailed explanation)

The seismic attribute has proven to be very efficient in characterizing geologic features such as MTDs and understanding their internal architecture from 3D data volume^3,12^. The MTDs are associated with coherent facies, possess variable dips and curvature. Moreover, they exhibit discontinuous geometry associated with the loss in frequency and energy. To capture these information, we have selected a suite of seismic attributes such as the similarity, DAV, energy, average frequency variance attributes etc. The readers can refer the works of authors^28,29^ for the definitions and mathematical equations pertaining to these attributes. It is crucial to parametrize these selected attributes so that they are able to arrest the MTD target from the entire seismic volume. The seismic attributes are defined using three vertical time windows (large: 80 ms, medium: 32 ms, and short: 24 ms) and 6 by 6 inline/xline step-outs (i.e., 6 traces in inline and 6 traces in xline directions) for efficient representation of the target.

### Example locations for training/testing (see the sub-section “Example Locations (train/test data)” in the supplementary note for detailed explanation)

The example locations are selected randomly along a few xlines and inlines, and the MTD-yes and MTD-no objects^28,29^ are defined based on seismic properties and geologic characteristics as described above (Fig. 5e). The MTD-yes objects are assigned with value of 1 and the MTD-no objects are associated with the value of 0 according to the binary classification rule. Around 755 objects (MTD-yes) and 745 non-objects (MTD-no) locations are labelled for training and testing. Thus, the object and non-object classes and the data points are almost balanced. The binary data (0,1) and seismic attributes at the picked or labelled locations are fed into the network for training and testing.

### Neural network design (see the sub-section “Neural Network Analysis” in the supplementary note for detailed explanation)

A fully connected MLP network (Figs. 5f and 8b) is designed for the computation of a hybrid or meta-attribute from a set of selected attributes that are related to the MTD. The MLP for this work consists of three distinct layers namely; the input, the hidden and the output layers. The seismic attributes and the binary numbers at the picked/labelled locations are fed into the input layer. The neurons of the hidden layer receive the data, where the information is summed up and further rescaled using an activation function. In this study we have used sigmoid function that is continuous, monotonically increasing, differentiable and further squashes the output in terms of 0 s and 1 s where, 0 s refer to the least probability of MTD and 1 s refer to the highest probability of the MTD.

The MLP network contains 5 neurons in input layer, 3 in hidden layer, and 2 in output layer, which are interconnected (Fig. 5f). Only 70% of the picked data are used for training in which the related seismic attributes are taken as input to compute the response lying between 0 and 1 using feed forward process^45,46,47,48^. The network parameters (rate of learning, momentum and most importantly the weights) are automatically adjusted iteratively based on back propagation algorithm^45,46,47,48^ to minimize the difference between the response and the train data (0,1). It is to be mentioned that the learning rate and momentum are optimally set to 0.01 and 0.25 respectively through several trials^28,29,39,44,49^. Since the process computes responses at remaining 30% locations (test data) also, the difference between the response and test data (0,1) is also calculated simultaneously to see the performance of the network by observing the nature of difference curve i.e. the decreasing trend of difference with iterations. Iterative neural training is continued till a minimum root-mean square (RMS) error between the response and train/test data is achieved such that a probability output is obtained at all picked locations^28,29,39,44^. The performance of the network is validated by a visual inspection with clipping the predicted meta-attribute over other seismic lines (for example, see Fig. 6). Once, satisfied with this validation, the network is made to run over the entire seismic cube such that the process of interpretation is automated and accelerated. We must state that the probability output with more than 0.75 threshold indicates the MTD target. This results into the generation of MTDC meta-attribute that automatically delimits the distribution of MTD in 3D space.

## Supplementary information

Supplementary information

## Data Availability

The data, used in applying this approach, was procured from the New Zealand Petroleum and Minerals, Ministry of Business, Innovation and Employment, New Zealand, [https://www.nzpam.govt.nz/] under certain restrictions and guidelines, and thus the data are not publicly available. However, the data can be procured for research with reasonable request and undertaking, and permission by New Zealand Petroleum and Minerals, Ministry of Business, Innovation and Employment, New Zealand [https://www.nzpam.govt.nz/].
