# Supplementary material for: A machine learning tool for interpretation of Mass Transport Deposits from seismic data
Source: Sci Rep. 2020 Aug 24;10:14134. doi: 10.1038/s41598-020-71088-6 (PMC7445243; doi:10.1038/s41598-020-71088-6)
Supplement: Supplementary file 1 — Supplementary information [file 41598_2020_71088_MOESM1_ESM.docx]

A machine learning tool for interpretation of Mass Transport Deposits from seismic data

Priyadarshi Chinmoy Kumar^a^ and Kalachand Sain^b, c,*^

Wadia Institute of Himalayan Geology, 33 GMS Road, Dehradun - 248001, India

[**^a^kumarchinmoy@gmail.com**](mailto:akumarchinmoy@gmail.com)**;** [**^b^kalchandsain7@gmail.com**](mailto:bkalchandsain7@gmail.com)**;** [**^c^director@wihg.res.in**](mailto:cdirector@wihg.res.in)

*Corresponding author**:** [**kalachandsain7@gmail.com**](mailto:kalachandsain7@gmail.com)

Supplementary Note

*Structural enhancement: structural filtering through extraction of dip-azimuth information*

Structural conditioning of seismic data maintains the lateral continuity of seismic events and provides enhanced subsurface structural image. In the present research, this is achieved by filtering the seismic volume of the Karewa prospect through a technique known as dip-steering, that results into a steering cube (or a dip-azimuth volume) (Tingdahl, 1999; Tingdahl and de Groot, 2003; Jaglan et al., 2015; Kumar and Mandal, 2017). The cube contains seismic dip and azimuth information at every sample position. The computation is achieved using the 3D Fourier Transform based dip algorithm proposed by Tingdahl (2003). The algorithm produces a stable dip-azimuth steering cube. The dip-azimuth were calculated at every position by transforming a sub-cube of 3x3x3 samples to the 3D Fourier domain and then the maximum dip was estimated with the help of a third-order polynomial curve fitting (Tingdahl, 2003) to the sub-cube around the sample of the highest energy in the Fourier domain. Then a search for the local maxima is made and the corresponding dip-azimuth to the local maxima is set as the output. The operation is carried throughout the seismic volume to generate the dip-azimuth volume, which at this stage is called as the ‘Detailed Steering Cube’ (DSC) and stores detailed structural information (Fig. S1a). The steering cube is a bit noisy and the overall structural trend may not be discernible. Hence, a coarser median filtering is applied to the DSC by a filtering step out i.e., inl: xrl: sample 5x5x5. The resulting steering cube is now called as the ‘Background Steering Cube’ (BSC), and contains overall dip trends of seismic reflectors and outlines the background structural information (Fig. S1b). The BSC is rather noise free as compared to the DSC, and is thus taken as the input for data enhancement through a process of structural filtering. The structural filtering is performed using a structure-oriented filter (SOF). The key objective of using the SOF is to differentiate between the dip-azimuth of seismic reflectors and overlying noises (Chopra and Marfurt, 2007). This removes not only the random noises but enhances the lateral continuity of seismic events (Höcker and Fehmers, 2002), and ultimately provides smooth and improved images of geological structures (Kumar and Mandal, 2017; Kumar and Sain, 2018, 2020).

For this, a statistical filter called as the Dip-Steered Median Filter (DSMF) is applied to the seismic volume using the pre-processed Steering Cube (SC) to obtain a smoothed seismic volume. The steering cube guides the median filter when it moves from one sample to the next within the seismic cube. The filter applies median statistics following the seismic dips within a dip-steered circle in which, the central amplitude is replaced by the median amplitude within the extracted circle. This results into an edge-preserving smoothing of the seismic data. The usefulness of a median filter is demonstrated in Fig. S2. To understand the use of a median filter, let us consider a range of amplitudes e.g., 0, 1, 0, 0, 0, 0, 1, 3. Now, upon applying a 3-point median filter to these amplitudes, the output becomes 0, 0, 0, 1, 1 (Fig. S2). The noise burst (amplitude value of 3) gets removed, edge remains preserved and no filter trails are introduced. However, increasing the filter size e.g., 5-point or 7 point shall over-smooth the data and would cause loss in amplitude. Thus, a care must be taken in this aspect. In this study a mild median filtering step-out of 3x3 is used to filter the seismic cube. The resulting DSMF data is a smoothed seismic cube in which the continuity of seismic reflections is improved and background random noises are suppressed (Fig. S3(a-b)). The DSMF seismic data is used for attribute extraction and further interpretation.


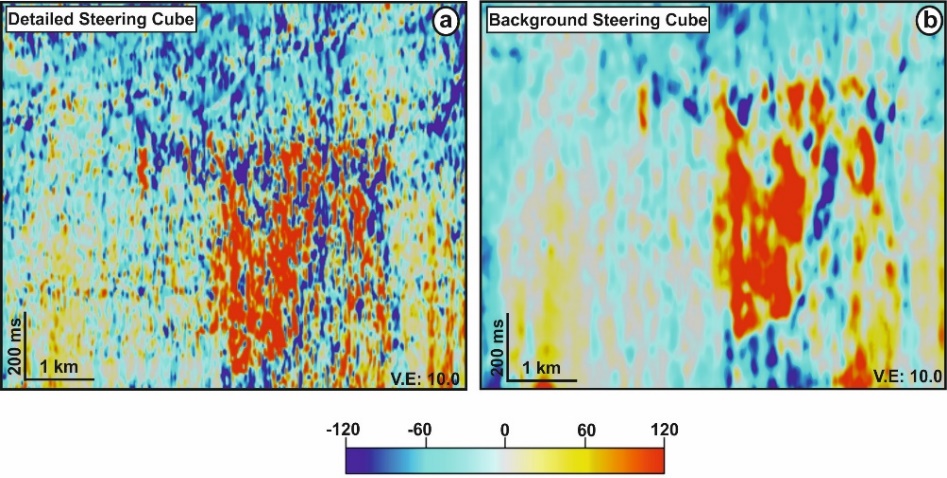


Figure S1: (a) Detailed steering cube outlining the structural trend for inline (IL 1177appears to be noisy; (b) Background steering cube outlining the overall structural trend. This noise-free steering cube is used for structural conditioning of the seismic volume. The color scale demonstrates the observed dip variations in microseconds per meter.


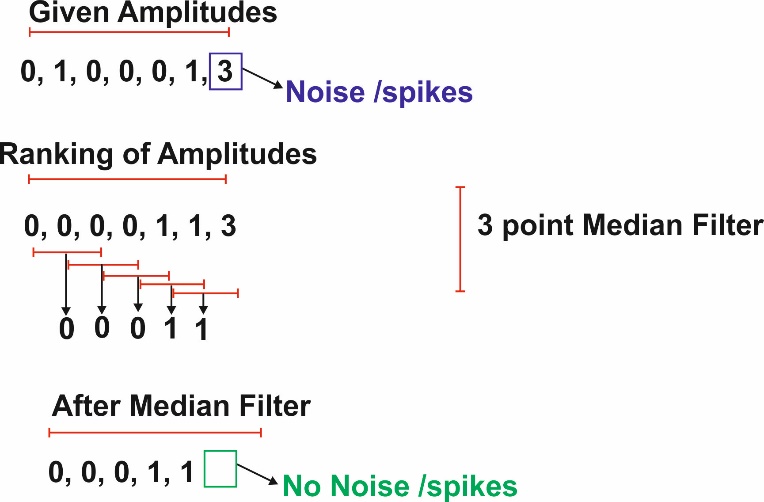


Figure S2: Operation of a median filter used for filtering of seismic data by a 3-point median filter that removes the noise bursts present into the data.


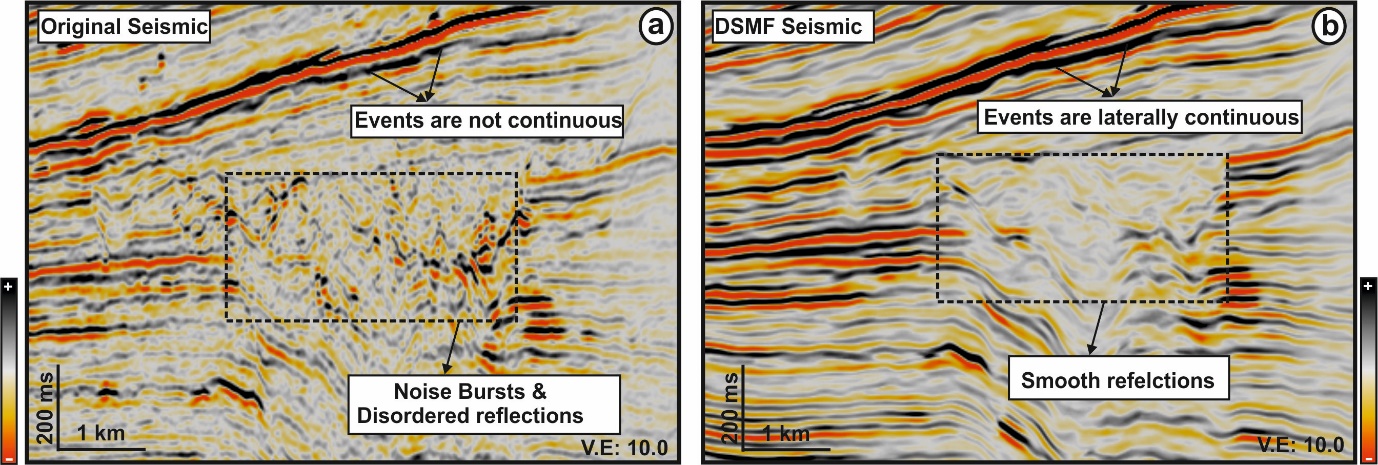


Figure S3: (a) Original Seismic section for IL 1177. Geologic features are masked by several noisy reflections. Seismic events are not laterally continuous and amplitude bursts are observed (within black dotted square); (b) DSMF seismic section for IL 1177 shows smooth and laterally continuous seismic reflections without any amplitude bursts.

*Initial Interpretation: The motivation to automate the approach using a machine*

Let us first step into understanding the MTD and its architecture in detail. This is demonstrated through a schematic representation that resembles the phenomena in the Karewa prospect, off New Zealand. Slope failure or slope instability results in the transportation of unconsolidated sediments – a process called as mass wasting, into deep water environments under the influence of gravitational force (Varnes, 1978; Posamentier and Kola, 2003; Omosanya, 2018). The voluminous mass that gets translated in such process, very often ranges from tens of cubic meters to about hundreds of thousands of cubic kilometres and further extends over an area of tens of millions of square kilometres (Moscardelli and Wood, 2008; Denne et al., 2013; Sobiesiak et al., 2018). During the failure of a submarine slope, material (or mass) flows from the regions of high gradient e.g., shelf-slope break and upper slope and gets translated downslope above a surface called as the basal shear surface (BSS) (Varnes, 1978; Martinsen, 1994; Bull et al., 2009) through a number of mass movement processes (Fig. S4a). The basal shear surface within these deposits gets developed due to progressive shear failure during sediment translation. Such submarine mass movement generates different depositional units that includes creep, slides, slumps and debri flows, which can be collectively termed as Mass Transport Deposits (MTDs). In seismic cross-sections, the top of MTD can be easily picked or recognised by an interpreter. However, mapping of BSS from the cross-sections becomes tedious as the reflector neither remains continuous, rather appears to be sheared. Identifying the top and bottom of MTD is crucial, as it defines the overall structural geometry. It is observed that several perplexing situations are encountered to define the base of the MTD (Fig. S4b). Internally, the MTD is associated with unconsolidated sediments. In seismic cross-section, the MTD is associated with disordered seismic reflections, loss in signal energy as well as frequency (Fig. S4b). For defining the overall 2D geometry of MTD, it becomes easier to some extent when an interpreter tries to analyse few 2D seismic transects. However, when the interpreter analyses a huge volume of 3D seismic data that consists of a number of xlines and inlines, it becomes painstaking to meticulously map top and bottom of the MTD to define its 3D structural architecture from seismic cube (Fig. S4b). Hence, if a machine is trained based on artificial intelligence, then the structural geometry of the MTD could be delineated at much ease without human intervention and with less time. This ideology motivates the present research to automate the process of delimiting the extension and distribution of MTDs from 3D seismic data.


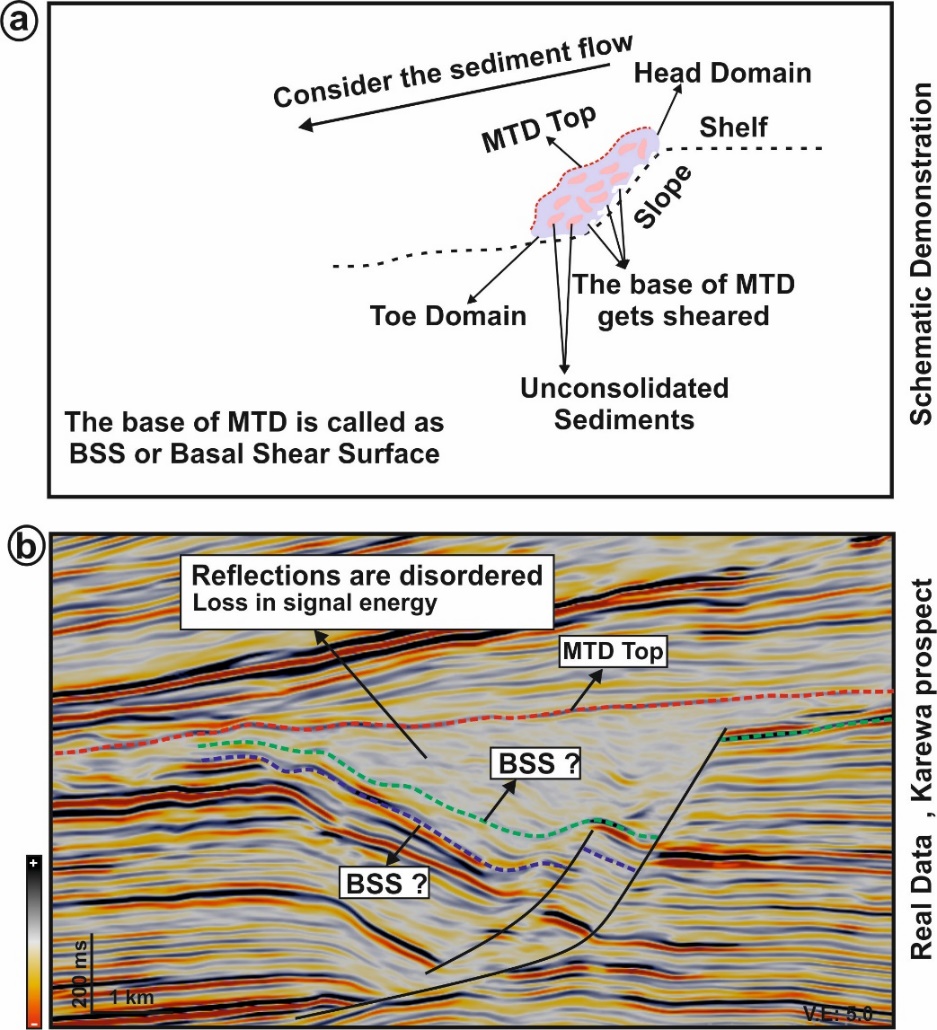


Figure S4: (a) Schematic diagram demonstrating the structure of MTD and associated processes; (b) Interpreted seismic section revealing the seismic characteristics of MTD. The upper bounding surface of the MTD is designated as MTD Top and bottom bounding surface as the basal shear surface (BSS). Several perplexing situation arises during the mapping of BSS, which has been indicated by question marks.

*From Seismic Attributes to Meta-attributes*

Seismic attribute analysis is a significant tool to enhance and isolate geologic features of interest from seismic data. Seismic attributes have the ability to capture anomalous response from the data and guide an analyst to interpret these responses in terms of subsurface geologic features (Bahorich and Farmer, 1995; Tingdahl, 2003; Chopra and Marfurt, 2007; Barnes, 2016; Kumar and Sain, 2018, 2020; Kumar et al., 2019a, b, c). However, use of individual attribute(s) for such interpretation suffers major drawbacks. To understand this, let us assume that an interpreter is trying to infer geologic faults from a given seismic cube. The interpreter immediately runs seismic attribute analysis to understand these discontinuous features. A discontinuity attribute (coherency or similarity) in principle highlights lateral changes in the seismic signal that includes sedimentary features (e.g., submarine channels, reefs, mass transport deposits etc.) as well as structural features e.g., faults. To honour the target of interest the interpreter then recombines other attributes with the discontinuity attribute such that signatures of sedimentary features get suppressed to some extent and those related to faults get enhanced. However, in such process of recombination, a complete isolation of geologic target (i.e., faults) may not be attained. Thus, an attribute hardly ever responds to a particular geologic object of interest (Kumar and Sain, 2018, 2020; Kumar et al., 2019 a, b, c) and struggles in discriminating geologic targets/objects of different origin. Hence, the output is a mixed set of geological information overlapped with noisy or unwanted features. For example, a discontinuity attribute may not map all the geologic faults that is observed by the interpreter from the data. Thus, the impact of geological non-uniqueness and non-completeness of an attribute keeps on changing based on its usage and is completely interpretation dependent (Brouwer et al., 2011; Kumar and Sain, 2018, 2020). If such is the case, how could this non-uniqueness or non-completeness of an attribute be alleviated? One possible solution may be perfecting or strengthening the algorithm or design of the attribute such that it can provide a unique answer. The other possible solution would be to recombine several standard attributes to obtain a more complex or robust attribute that can completely isolate the geologic target of interest from the surrounding (Aminzadeh and de Groot, 2006; Kumar and Sain, 2018). This ideology has given birth to the concept of meta-attributes that are obtained by logically combining a set of suitable attributes through artificial neural networks (Kumar and Sain, 2018, 2020; Kumar et al., 2019 a, b, c). The amount of refinement to be needed or the choice of the best method to be adopted completely depends on the interpretation problem and the desired accuracy of the output.

*The MTD Cube Meta-attribute: Process and Design*

The MTD Cube meta-attribute can be logically designed using a combination of several suitable attributes through artificial neural networks.

Attribute Selection

It is important to find out the suitable attributes and their choice of selection. The successful approach to select a set of suitable attributes for MTD interpretation is that they are able to define the MTDs from the data. We stress that the interpreter should have a sound knowledge on the geologic target of interest that is aimed to be interpreted. In the present research, the target is the Karewa MTD zone from seismic cube. To begin the selection, it is crucial to understand the characteristics of MTD from seismic data.

1. MTDs are considered to be the intervals of chaotic or highly disrupted seismic facies (Fig. S4(b)).
2. As MTDs undergo complex internal deformation and their bottom appears to be sheared, there exists limited lateral correlation of reflectors showing low similarity between seismic traces.
3. MTDs are associated with the loss of energy and frequency within, as they are mainly made up of unconsolidated sediments.
4. Due to complex deformation, the sediments within MTDs are disorderedly arranged, which lead to variable dips and azimuth.

The above mentioned characteristic properties makes the set of geometric and physical seismic attributes e.g., similarity/coherency, dip, azimuth, energy, frequency to be good choice for enhancing the interpretation of MTDs from seismic data. Being governed by these facts, in the present study, attributes e.g., similarity, dip angle variance, average frequency variance and energy are grouped as set of suitable/standard input seismic attributes that can be combined to generate complex attribute or the meta-attribute. After selecting these attributes, it is crucial to parametrize them so that they are able to capture the MTD units from seismic data volume. The seismic attributes are extracted using three vertical time windows (large: 80 ms, medium: 32 ms, and short: 24 ms), and 6x6 inline and crossline step-outs.

Example Locations (Train/Test Data)

For this, an interpreter provides example locations (x, y, z) to differentiate between the target locations (MTD-yes i.e., the zone of interest) and non-target locations (MTD-no i.e., all zones surrounding the MTD unit or non-zone of interests) from the seismic cube (Fig. S5).


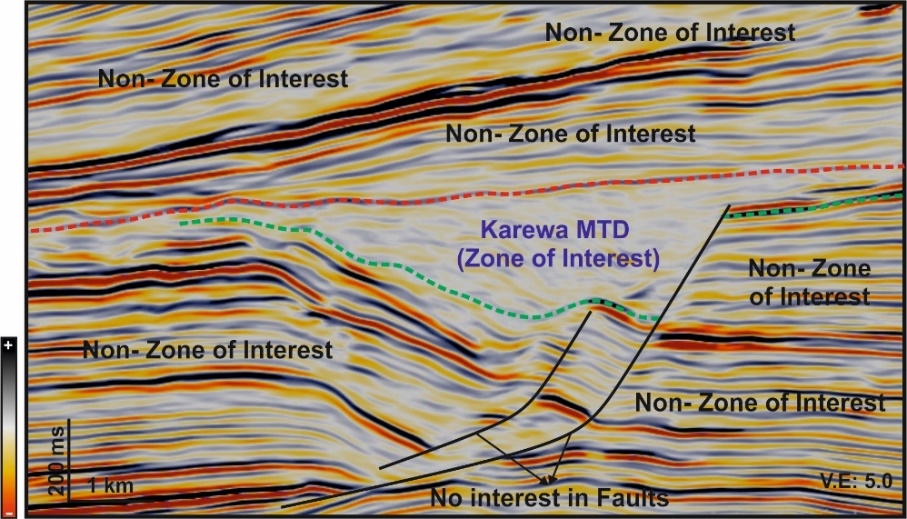


Figure S5: Seismic section displaying the target and non-target zones. The zone of interest is the Karewa MTD and the areas surrounding the MTD are the non-interest zones. The top of MTD is marked by red dotted line, whereas the BSS is indicated by green dotted line. Black solid lines represent faults.

These locations are assigned over key seismic lines that are randomly chosen from seismic cube. Ambiguous areas where the interpreter is not sure for the presence of the target or the areas that are devoid of target are avoided. The process of assigning example locations is described in detail through the following demonstration (see Fig. S6).

1. In the first step, the interpreter randomly examines the seismic lines for the presence of MTD. Again, we would like to stress here that an analyst must be able to identify the targets/objects (Fig. S6a-b).
2. In the second step, the interpreter further investigates the seismic characteristics of the target over these lines with the help of attributes responses. (Fig. S6c).
3. Combining the first and second steps, the interpreter finally decides the appropriate locations that should be assigned for the network to learn (Fig. S6d).
4. Neural training is then executed over these seismic lines to compute the meta-attribute.
5. Once, being satisfied with the performance of the neural model, the network is made to scan over the entire seismic cube to generate the hybrid attribute, defined as the MTD cube or MTDC meta-attribute.

The entire step is detailed in Fig. S6. It is observed that the MTD zone is characterized by disrupted and chaotic reflections and is internally deformed. Apart from MTD we also find several other geological features e.g., faults, depositional beds etc. Since our interest is to delineate MTD, it should remain isolated from other zones for the success of attribute combinations. The result of similarity attribute brings out all possible low similar values representing the faults, stratigraphic packages and the MTD zone. Thus, the correct example location should be the MTD-yes zone and all other false location should be the MTD-no zone. Strictly speaking, example locations mean the target and non-target zones within the given seismic data. The MTD-yes objects are assigned with value of 1 and the MTD-no objects are associated with the value of 0 according to the binary classification rule.


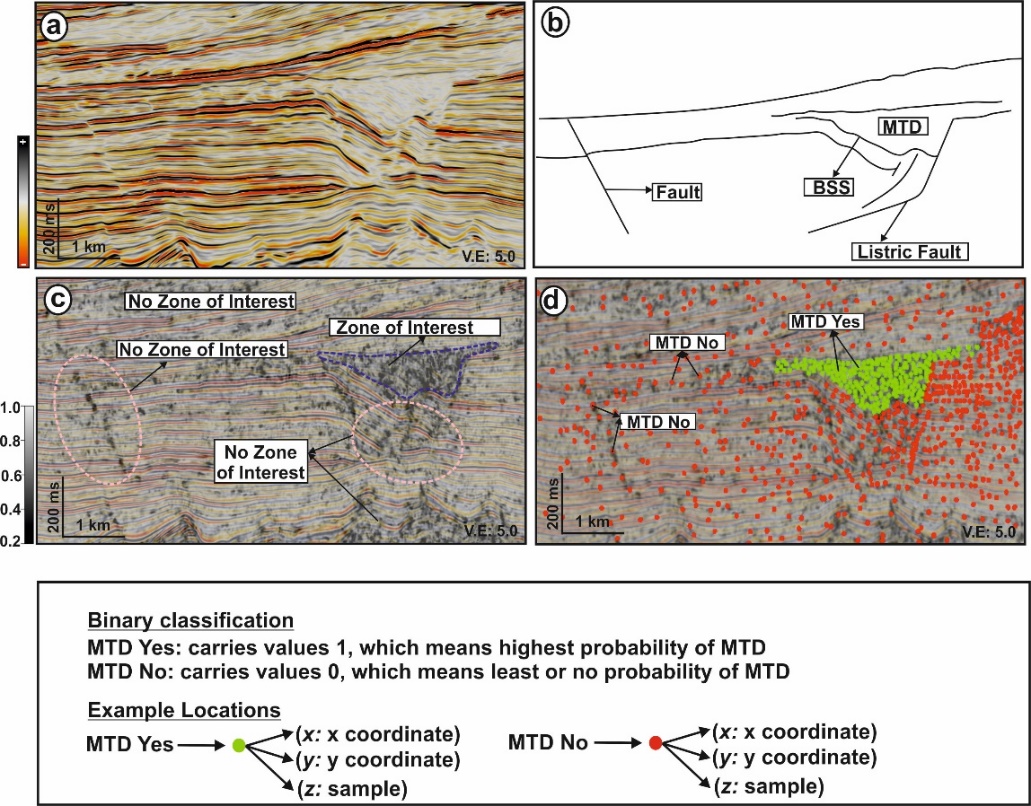


Figure S6: (a) Uninterpreted seismic section from Karewa 3D prospect; (b) Sketches of interpretation perceived after examining the seismic section in (a); (c) Seismic section co-rendered with the computed similarity attribute demonstrating the interest and non-interest zones; (d) Assigned example locations. The green dots refer to MTD-Yes location and red dots refers to MTD-No location.

Neural Network Analysis

The neural network chosen for designing the meta-attribute is a fully connected multi-layer perceptron (MLP) network (Kumar and Sain, 2018, 2020; Kumar et al., 2019a, b, c). The simple and most widely used ANN is the Multi-layer perceptron (MLP) network, the architecture of which is based on different layers namely: the input layer, the hidden layer and the output layer. Each layer consists of different neurons which are associated with a synaptic weight that tries to strengthen the connection. The data fed into the network, flows from left to right (i.e., from the input to the output). Nodes in the input layer simply pass the information to the hidden layer, where the net input is passed through the activation/ performance function. The final output is computed by summation over the output nodes in the hidden layer multiplied by their corresponding weights. When all the nodes in a layer are connected to all the nodes in the next layer through weights, the network is called as the Multi-layer perceptron. When the data flows between the layers, it is called as the feed-forward process. Optimization of the network is carried out by back-propagation algorithm (Rosenblatt, 1962; Rumelhart et al., 1995) that attempts to minimize the error between the predicted network result and the known output by automatically adjusting the connection weights and other network parameters e.g., learning rate and momentum.

In this study, the hand-labelled data at picked locations is split into 70/30% chunks for training and testing respectively. For training, the related attributes have been taken as input to compute the response lying between 0 and 1 using the feed forward process (Rosenblatt, 1962; Poulton, 2001, 2002). The network parameters (learning rate, momentum and weights) are automatically adjusted iteratively based on back propagation algorithm (Rosenblatt, 1962; Poulton, 2001, 2002) to minimize the difference between the response and the train data (0,1). Since the process computes the responses at the remaining 30% locations (test data), the difference between the response and the test data (0,1) is also calculated simultaneously to see if the neural model is trained properly for prediction by observing the behaviour i.e. the decreasing trend (Fig.S7) of difference with iterations. Iterative neural training is continued till a minimum normalized root-mean square (nRMS) error and misclassification percentage between the computed response and train and test data (0,1) is achieved such that a probability output is obtained at all picked locations. This has been described in the next-to-next paragraph.


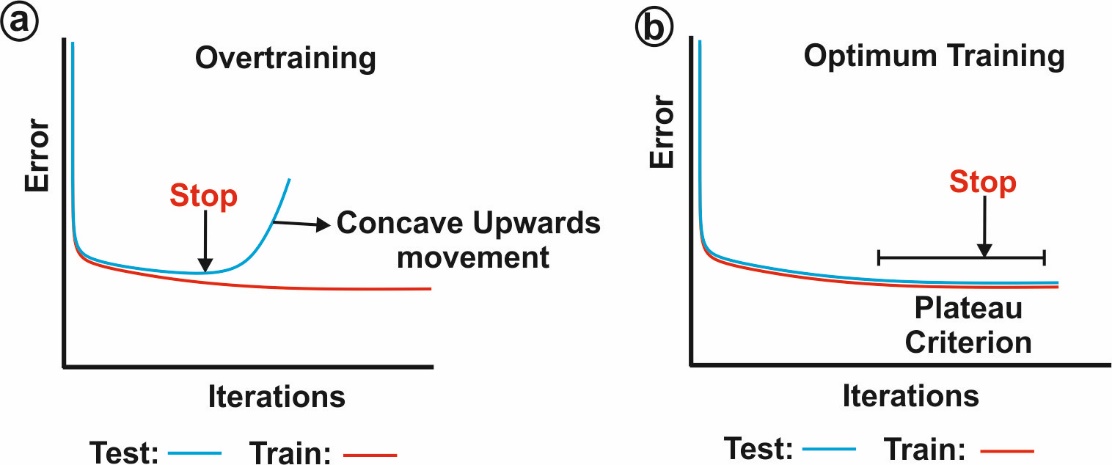


Figure S7: (a) Schematic representation of error curves demonstrating the overtraining situation. Neural training should be stopped immediately when the test curve starts showing concave upward pattern; (b) Schematic representation of error curves demonstrating the optimum training situation. This is achieved when both the error curve undergoes a smooth decay and becomes plateau with the increase in iterations.

Most importantly, the MLP network possess two important features e.g., abstraction (the ability to extract relevant features from the input pattern and discard the irrelevant ones) and generalization (recognize the input patterns which are not the part of the training set). During the training phase, it is crucial to identify a proper stopping criterion in-order to escape from overtraining, which occurs when the neural network find relations in the training examples are not universal. Suppose that while assigning example locations to few seismic lines in the cube, the interpreter is unable identify correct MTD zone from the data and assigns MTD-yes as the zone that is devoid of the features. At this point the training becomes non-universal, meaning the network will fail to judge whether these locations belong exactly to MTD or partially to MTD. Hence, the network at this point is said to be overtrained as it is unable to find a universal solution to such a perplexing situation. An optimum training (Fig.S7) is generally obtained when the error within the train and test data sets reaches a plateau (Kumar and Sain, 2018, 2020; Kumar et al., 2019a, b, c).

The performance evaluation of neural training in the present study is tracked through two different parameters namely the normalized root-mean squared (nRMS) error and the misclassification percentage. The nRMS error is computed from the RMS error between the targeted ($t_{i}$) and computed ($c_{i}$) values for $i$ ranging from 1 to n, which is given as (Kumar et al., 2019 a,b,c):

$RMS= \sqrt{\frac{1}{n}} \sum_{i=1}^{n} {(t_{i}-c_{i})}^{2}$ ………………………………(1)

$normalized RMS= RMS/\sqrt{\frac{1}{n}} \sum_{i=1}^{n} {(t_{i}-mean)}^{2}$ ……………………………(2)

where, the mean is given as:

$mean = \frac{1}{n} \sum_{i=1}^{n} t_{i}$ …………………………………………..(3)

The nRMS error curve demonstrates the overall error on the train and test data sets, with a scale ranging from 0 to 1, where 0 corresponds to no error and 1 corresponds to highest error. It has been demonstrated that the lower the nRMS, the better the neural outcome is (Kumar and Sain, 2018, 2020; Kumar et al., 2019a, b, c). The other benefit is that error performance can be observed from a single graph display.

The second performance evaluation parameter is the misclassification percentage. It is a quality control parameter to understand the wrong predictions made during the classification. To have a control over this accuracy, it is good enough to create a truly representative distribution of the observations for each class (i.e., target and non-target zones). The classification percentage is defined as the ratio of correct predictions to that of the total number of predictions.

$Classification \left( \% \right)= \frac{Number of correct predictions}{Total numebr of predictions}x 100$ ……….(4)

The misclassification percentage is defined as the ratio of wrong predictions to that of the total number of predictions.

$Misclassification \left( \% \right)= \frac{Number of wrong predictions}{Total numebr of predictions}x 100$ ……..(5)

To judge this performance evaluation parameters of the intelligent neural model, we follow a general rule i.e., lower the nRMS, lower would be the misclassification percentage (Kumar and Sain, 2018, 2020; Kumar et al., 2019a, b, c). In the present study, the computed MTD Cube neural model is evaluated based on these parameters. The output is a probability output, called as the MTD Cube meta-attribute, which carries values ranging from 0 to 1. An optimum color scale is used in such a way that the maximum probability i.e., values closer to 1 are displayed and visualized, and those pertaining to values closer to 0 i.e., the least probability are made transparent. After making a quality check of the neural model over randomly selected a few seismic lines, the network is made to run over the entire seismic cube to limit human intervention and speed up the interpretation task. This results into the so-called MTDC meta-attribute. In this process of neural network analysis, the role of an analyst is to provide only example locations over a small segment of data and logically confirm the quality of the intelligent model. Once satisfied on the performance by visually inspecting the prediction over a few un-interpreted seismic sections, the model is ready to run over the entire seismic cube to fasten up the interpretation. This process of neural learning is called as a supervised neural learning where an engineered machine tries to follow teachings offered by the intelligent machine i.e., the human brain and generates an optimized output to the assigned problem.

**References**

Aminzadeh, F., & De Groot, P. Neural networks and other soft computing techniques with applications in the oil industry. EAGE Publications (2006).

Bahorich, M. & Farmer, S., 1995. 3-D seismic discontinuity for faults and stratigraphic features: The coherence cube. Lead. Edge. 14. 1053-1058 (1995).

Barnes, A. E. (Ed.). Handbook of poststack seismic attributes. Society of Exploration Geophysicists (2016).

Brouwer, F., Tingdahl, K. & Connolly, D. A Guide to the Practical Use of Neural Networks. Annual Gulf Coast Section SEPM Foundation Bob F. Perkins Research Conference, Houston, Texas (2011).

Bull, S., Cartwright, J. & Huuse, M. A review of kinematic indicators from mass-transport complexes using 3D seismic data. Mar. Pet. Geol. 26, 1132–1151 (2009).

Chopra, S. & Marfurt, K.J. Seismic attributes for prospect identification and reservoir characterization. Society of Exploration Geophysicists and European Association of Geoscientists and Engineers (2007).

Denne, R. A., Scott, E. D., Eickhoff, D. P., Kaiser, J. S., Hill, R. J., & Spaw, J. M. Massive Cretaceous-Paleogene boundary deposit, deep-water Gulf of Mexico: New evidence for widespread Chicxulub-induced slope failure. Geology, 41(9), 983-986 (2013).

Höcker, C. & Fehmers, G. Fast structural interpretation with structure-oriented filtering. The Lead. Edge., 21, 238-243 (2002).

Jaglan, H., Qayyum, F. & Hélène, H. Unconventional seismic attributes for fracture characterization. First Break, 33, 101-109 (2015).

Kumar P.C. & Mandal, A. Enhancement of fault interpretation using multi-attribute analysis and artificial neural network (ANN) approach: A case study from Taranaki Basin, New Zealand. Exploration Geophysics, 49(3), 409-424 (2017).

Kumar, P.C. & Sain, K. Attribute amalgamation-aiding interpretation of faults from seismic data: An example from Waitara 3D prospect in Taranaki basin off New Zealand. Journal of App. Geophy., 159, 52-68 (2018).

Kumar, P. C., & Sain, K. Interpretation of magma transport through saucer sills in shallow sedimentary strata using an automated machine learning approach. Tectonophysics, 789, 228541, (2020).

Kumar, P.C., Omosanya, K. O. & Sain, K. Sill Cube: An automated approach for the interpretation of magmatic sill complexes on seismic reflection data. Mar. & Pet. Geol., 100, 60-84 (2019a).

Kumar, P.C., Sain, K. & Mandal, A. Delineation of a buried volcanic system in Kora prospect off New Zealand using artificial neural networks and its implications. J. of App. Geophy., 161, 56-75, (2019b).

Kumar, P.C., Omosanya, K. O., Alves, T.M. & Sain, K. A neural network approach for elucidating fluid leakage along hard-linked normal faults. Mar. & Pet. Geol., 110, 518-538 (2019c).

Martinsen, O.J. Mass movements, in: Maltman, A. (Ed.), The Geological Deformation of Sediments. Chapman and Hall, pp. 127–165 (1994).

Moscardelli, L., Wood, L. New classification system for mass transport complexes in offshore Trinidad. Basin Res. 20, 73–98 (2008).

Omosanya, K.O. Episodic fluid flow as a trigger for Miocene‐Pliocene slope instability on the Utgard High, Norwegian Sea. Basin Res., 30, 942-964 (2018)

Posamentier, H.W. & Kolla. Seismic geomorphology and stratigraphy of depositional elements in deep-water settings. J. Sediment. Res. 73, 367–388 (2003).

Poulton, M.M. Computational neural networks for geophysical data processing. Elsevier (2001)

Poulton, M.M. Neural networks as an intelligence amplification tool: A review of applications. Geophysics, 67(3), 979-993, (2002).

Rosenblatt, F. Principles of neurodynamics. perceptrons and the theory of brain mechanisms (No. VG-1196-G-8). Cornell Aeronautical Lab Inc Buffalo NY (1962).

Rumelhart, D.E., Durbin, R., Golden, R. & Chauvin, Y. Backpropagation: The basic theory. Backpropagation: Theory, architectures and applications, pp.1-34 (1995)

Sobiesiak, M.S., Kneller, B., Alsop, G.I. & Milana, J.P. Styles of basal interaction beneath mass transport deposits. Mar. Pet. Geol. 98, 629–639 (2018).

Tingdahl, K.M. Improving seismic detectability using intrinsic directionality, Paper B194. Earth Science Centre, Goteberg University (1999).

Tingdahl, K. M. & de Groot, P. F. Post-stack dip and azimuth processing, J. Seis. Explor, 12, 113-126 (2003).

Tingdahl, K. M. Improving seismic chimney detection using directional attributes, in: Nikarvesh, M., Aminzadeh, F., Zadeh, L.A, (eds.) Soft Computing and Intelligent Data Analysis in Oil Exploration, Developments in Petroleum science, Elsevier, Amsterdam. 157-173 (2003).

Varnes, D.J. Slope movement types and processes., Schuster, R.L., Kruse, R.J. (Eds.), Landslides, Analysis and Control.: Special Report, 176 (1978). National Academy of Sciences, Washington.
